# Microsurgical Wiltse Paraspinal Approach Using a Low-Budget Exoscope

**DOI:** 10.7759/cureus.25858

**Published:** 2022-06-11

**Authors:** Manuel de Jesus Encarnacion Ramirez, Rossi E Barrientos Castillo, Renat Nurmukhametov, Medet Dosanov, Nikita Tolokonnikov, Edwin Bernard, Ibrahim E Efe

**Affiliations:** 1 Department of Neurosurgery, People's Friendship University, Moscow, RUS; 2 Division of Spine Surgery, Russian Academy of Sciences, Moscow, RUS; 3 Department of Neurosurgery, Charité - Universitätsmedizin Berlin, Berlin, DEU

**Keywords:** exoscope, low-cost, transforaminal lumbar interbody fusion, minimally-invasive spine surgery, wiltse approach, paarspinal approach

## Abstract

Access to microneurosurgical care in low- and middle-income countries remains limited mainly due to a lack of equipment. High purchasing and maintenance costs hinder the use of operating microscopes in low-resource facilities. The authors present an improved version of their previously introduced low-cost exoscope to achieve high magnification and illumination in low-resource environments. The setup included a 48-megapixel two-dimensional digital microscope camera, a wide field C-mount lens, ring light, and a two-link cantilever with a screw terminal. The surgical field was projected to a portable 17.3-inch 2K resolution monitor. Ten patients underwent exoscope-assisted transforaminal lumbar interbody fusion via the Wiltse paraspinal approach. The simple construction allowed a fast and intuitive preoperative setup. The in-plane switching type display provided a clear and bright image regardless of the viewing angle. The two-link arm of the cantilever allowed smooth positioning of the camera, overcoming the cumbersome up and down movements needed to zoom in and out with the previous prototype. Industrial microscope cameras are effective low-budget alternatives to conventional operating microscopes in lumbar microdiscectomy. The improved system is superior compared to the authors' previous prototype with regard to affordability, image quality, and adjustability of position and angle.

## Introduction

Only a quarter of the world's population has access to microneurosurgical facilities within a range of two hours. High acquisition and maintenance costs hinder the use of operating microscopes and exoscopes in low-resource environments [1]. The authors recently described a simplified exoscope composed of industrial digital microscope parts. They reported sufficient magnification and illumination in 13 spinal and three cranial surgeries. Yet, their setup showed considerable limitations including the cumbersome manual adjustment of zoom and camera position [2]. Here, the authors introduce a new version providing smoother adjustability at only half the cost of the initial prototype. The new system was tested in a cohort of 10 patients undergoing the microsurgical Wiltse paraspinal approach for transforaminal lumbar interbody fusion (TLIF) surgery.

## Technical report

Device properties

**Figure 1:**
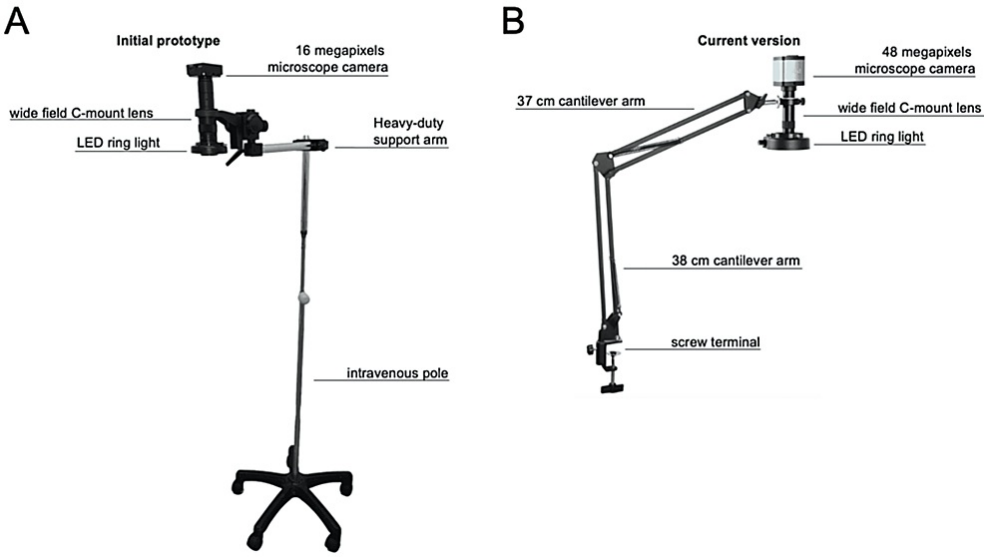
(A) The previously published prototype was composed of a 16-megapixel industrial microscope camera, a wide field C-mount lens and an LED ring light attached to an intravenous pole via a heavy-duty support arm. To zoom in and out, the support arm was loosened and moved down or up along the pole, respectively. (B) The improved version of the low-cost exoscope includes a 48-megapixel digital microscope camera and a two-link cantilever that can be attached to the operating table via a screw terminal. The two-link arm allows seamless adjustment of zoom and angle with a wide range of motion.

The authors purchased a 48 megapixels light-weight video microscope camera and a wide-field industrial C-mount lens (Eakins, Shenzhen Huaxin Electronic Trading Limited Company (Co., Ltd.), Shenzhen, China). An LED ring light provided shadow-free focused illumination and adjustable brightness. A cantilever with a two-link arm allowed for manual adjustment of the camera position and angle with a wide range of motion (Eakins, Shenzhen Huaxin Electronic Trading Co., Ltd., Shenzhen, China). The cantilever was attached to the operating table through a screw terminal. The surgical field was projected to a portable 2K high-resolution in-plane switching (IPS) monitor placed at eye level at a 100- to 120-cm distance (Porpoise, China). The total cost of the present setup was roughly US$ 350 (Table 1). The authors' previously published setup was purchased at a total cost of US$ 750 (Figures 1A, 1B) [2].

**Table 1:** Exoscope components and their specifications, brands and costs. LED = light-emitting diode; SD = secure digital; HDMI = high-definition multimedia interface; IPS = in-plane switching; GB = gigabytes

Component	Specifications	Brand	Cost
Microscope camera	48 megapixels high resolution image sensor, 1920x1080 pixels and 60 frames per second video output, automatic white balance	Eakins by Shenzhen Huaxin Electronic Trading Co., Ltd. (Shenzhen, China)	US$ 87
Wide field industrial C-mount lens	Objective magnification power by 0.13 – 2x (up to 130x on the monitor)	Eakins by Shenzhen Huaxin Electronic Trading Co., Ltd. (Shenzhen, China)	US$ 19
Cantilever	Two-link arm (37 cm and 38 cm)	Eakins by Shenzhen Huaxin Electronic Trading Co., Ltd. (Shenzhen, China)	US$ 38
LED ring light	Intense and focused shadow-free white light, adjustable brightness	Eakins by Shenzhen Huaxin Electronic Trading Co., Ltd. (Shenzhen, China)	US$ 11
Portable monitor	17.3” 2K resolution IPS type panel	Porpoise (China)	US$ 193
Micro SD card	16 GB storage	QEEDNS (China)	US$ 3
HDMI cable		Unknown	US$ 2
			Total cost: US$ 353

**Figure 2:**
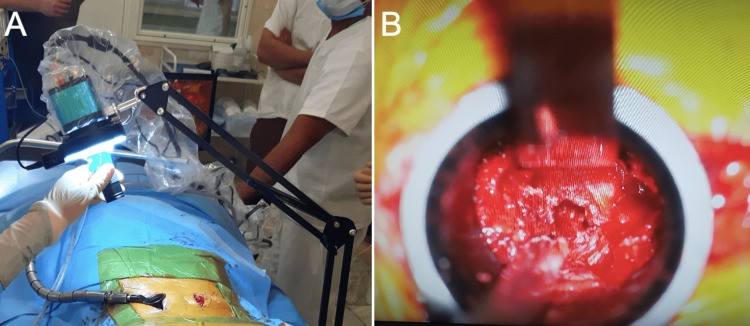
(A) Cables and microscope cameras were covered with sterile drapes. The remaining components were sterilized with ethylene oxide. The cantilever was attached to the contralateral side of the operating table. The ring light was secured at the upper edge of the microscope tube. The lower half of the microscope tube allowed a firm grip to adjust the camera's position and angle. (B) The exoscope provided sufficient magnification and illumination of the surgical field despite a narrow surgical corridor.

The cantilever, LED ring light, and C-mount lens were sterilized with ethylene oxide. The microscope camera and cables were covered with sterile drapes (Figures 2A, 2B).

Patient characteristics

The exoscope was tested in six female and four male patients. The mean age was 52.2 (39-70) years. One patient underwent a TLIF at the L3-L4 segments, six at the L4-L5 segments, and three at the L5-S1 segments. All patients presented with lower back pain and radiculopathy with mild to moderate sensorimotor deficits. Individual patient consent was obtained prior to enrollment.

Surgical technique

**Figure 3:**
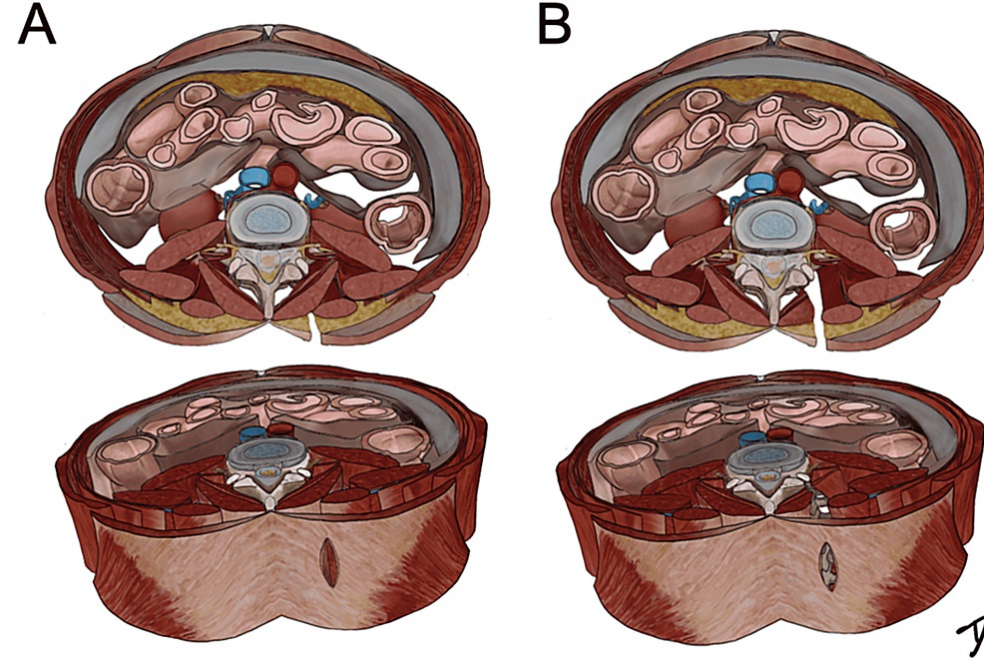
(A) The Wiltse approach begins with a paravertebral incision 4 cm lateral to the midline. Using blunt dissection, the natural corridor between the multifidus and longissimus muscles is opened. (B) The junction between the facets and the transverse process is exposed.

The Wiltse approach is an old technique for achieving a minimally invasive corridor to the posterolateral spine. Minimal intraoperative bleeding, short hospital stay, and low infection rates render the Wiltse approach an elegant alternative to the standard midline posterior approach [3]. Surgery was performed under general anesthesia. Patients were operated on in prone positions. Intraoperative C-arm fluoroscopy helped exactly locate the affected segments. Two paravertebral incisions were made 4 cm lateral to the midline. The thoracolumbar fascia was opened longitudinally roughly 3 cm lateral to the spinous processes using monopolar cauterization. The medial multifidus muscle was mobilized away from the lateral longissimus muscle to open a natural corridor to the junction between the facets and the transverse process (Figures 3A, 3B).

**Figure 4:**
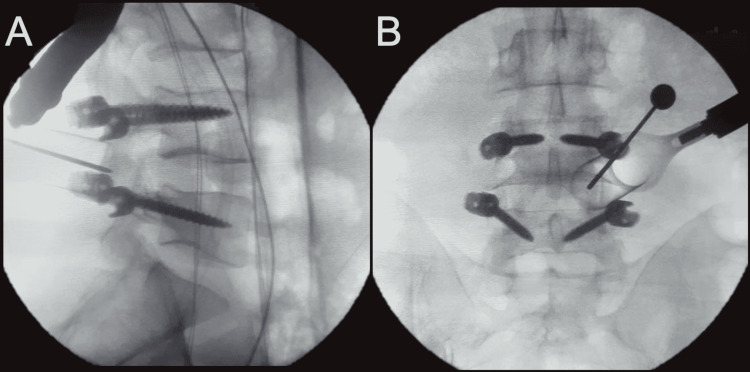
(A) Sagittal and (B) coronal x-ray images of the lumbosacral spine after bilateral pedicle screw placement at the L4/L5 segments in a 44-year-old male patient.

A Caspar retractor system for minimally invasive spine surgery was installed and a Penfield dissector no. 4 was inserted for radiological confirmation of the correct segment. Pedicle screws were inserted on both sides (Figures 4A, 4B). A tubular retractor with a 22 mm diameter was inserted.

The authors proceeded under microscopic vision using the low-cost exoscope (Video 1). The cantilever was mounted to the contralateral side of the operating table. A laminectomy and facetectomy were performed by removing the inferior articular process and the superior third of the superior articular process.

**Video 1:** High-resolution footage of the surgical field as captured by the low-cost exoscope, following removal of the right inferior facet in a 44-year-old male patient.

A flavectomy was performed to expose the dural sac and intervertebral disc. Following microsurgical discectomy, an interbody cage filled with bone autograft was placed and screws were connected through rods. The exoscope-assisted stage of the surgery accounted for approximately a quarter of the total operation duration (Table 2). Pain relief was achieved in all patients. Nine showed full sensorimotor recovery, one showed partial motor recovery at a six-week follow-up. There were no intraoperative complications and the mean blood loss was 198 (110-260) mL.

**Table 2:** Overview of demographic, intraoperative, and clinical data. No. = number; M = male; F = female

Patient No.	Age, sex	Segments	Exoscope usage in minutes (percentage of total surgery duration)	Intraoperative blood loss in ml	Postoperative hospital stay in days	Surgical outcome
1	43, M	L3-L4	35 (26%)	188	3	Pain relief, full sensorimotor recovery
2	47, F	L4-L5	32 (25%)	210	3	Pain relief, full sensorimotor recovery
3	63, F	L4-L5	25 (21%)	240	4	Pain relief, full sensorimotor recovery
4	48, F	L4-L5	26 (20%)	180	3	Pain relief, full sensorimotor recovery
5	44, M	L4-L5	27 (25%)	260	3	Pain relief, full sensorimotor recovery
6	70, F	L4-L5	30 (25%)	230	3	Pain relief, full sensorimotor recovery
7	39, F	L4-L5	30 (23%)	110	3	Pain relief, full sensory recovery, partial motor recovery
8	61, M	L5-S1	36 (28%)	144	3	Pain relief, full sensorimotor recovery
9	57, M	L5-S1	28 (24%)	190	3	Pain relief, full sensorimotor recovery
10	50, F	L5-S1	31 (25%)	230	4	Pain relief, full sensorimotor recovery

User experience

**Figure 5:**
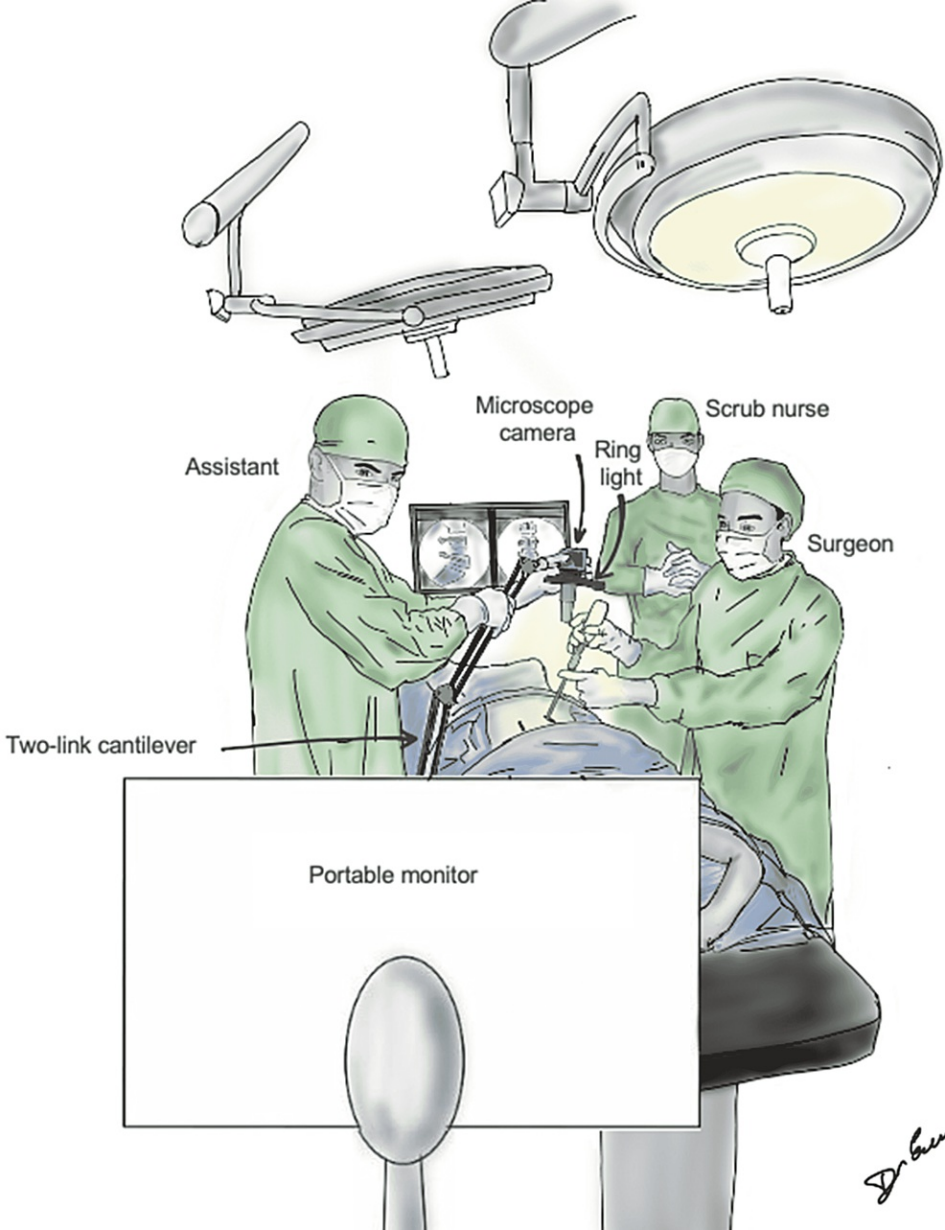
Graphical illustration of the operating room setup during the exoscope-assisted phase of the surgery.

The simple construction of the exoscope allowed intuitive preoperative preparation in under five minutes. The screw terminal at the base of the cantilever enabled fast installment to the operating table when proceeding to the microsurgical stage of the surgery. Alternatively, the cantilever could be attached to an intravenous pole like the previous prototype. The surgical field was projected to a portable 17.3” 2K high-resolution monitor. Albeit small, the IPS-type panel provided a clear and bright image regardless of the viewing angle. This allowed the assistant surgeon and the nurse to follow the surgery on the same screen (Figure 5).

This was particularly beneficial for teaching younger staff. Operative footage could be recorded although our 16 GB micro SD card provided storage space for a maximum of two surgeries only. Video files were thus transferred to a hard disk following each surgery. The lack of three-dimensional (3D) vision remained a main limitation in this new prototype, too. The ring light achieved sufficient illumination with no need for additional overhead lamps. Lighting was best when the camera was set at a working distance of 40 to 60 cm. The authors did not experience any significant glare issues. The two-link arm of the cantilever allowed smooth positioning of the camera, overcoming the cumbersome up and down movements needed to zoom in and out with the previous prototype. The small size of the setup allowed switching between microscopic and macroscopic views without having to move the device away from the surgical field. The overall user experience was superior compared to the initial system. 

## Discussion

The authors previously encountered three main limitations when working with the initial prototype: cumbersome adjustment of the zoom, insufficient lighting, and lack of depth perception [2]. Similar to the previous model, the microscope camera presented in this technical report did not allow zooming in a gradual but only stepwise manner with too large, hence impractical intervals. Previously, the support arm had to be loosened, moved down, and secured at the desired height. The present system relies on a two-link cantilever similar to those of desk lamps. This allowed seamless single-handed up and down movements of the camera. Further, the LED ring light was attached to the upper end of the C-mount microscope tube so the lower end of the tube could be used as a handle. These features minimized the disruptions of the intraoperative workflow which the group experienced with the initial prototype.

The authors had previously used a 55” LED monitor as they felt more confident looking at a large screen [2]. In the present setup, they chose a small but 2K IPS type monitor. This display type allows clear and bright visual quality regardless of the viewing angle which made the new setup particularly attractive for teaching.

The lack of depth perception remained a key limitation. This is a typical drawback of commercially available exoscope systems [4]. Low-budget high-definition 3D cameras built for recreational use may hold promise for achieving 3D vision at a low cost.

The prices of exoscope systems available on the market range from $250,000 to $1,500,000 [5]. The setup presented in this technical report was purchased at only 350 US$. The authors achieved a 53% cost reduction compared to the previous prototype [2].

This study included only 10 patients and was limited to one surgical technique. The Wiltse approach was a suitable procedure to test the device due to its standardized workflow and the narrow hence challenging surgical corridor. The authors achieved satisfying results in all ten cases with no considerable delay in surgical duration. Comparative studies may help assess how the low-budget exoscope performs compared to commercially available binocular microscopes or exoscopes. Further, larger patient cohorts and approaches other than those to the lumbar spine are needed to prove this low-cost technology effective for a broader use in neurosurgery. Thus far, the device has only been tested by the authors and their department. It has yet to be critically appraised by independent specialists. The authors hope their work will encourage colleagues to prototype similar low-cost solutions to help expand microneurosurgical care in low-resource settings.

## Conclusions

Industrial microscope cameras are a low-cost alternative to conventional surgical visualization systems in low-resource environments. The presented low-cost exoscope proved effective in lumbar microdiscectomy as part of the Wiltse paraspinal approach. It is superior compared to the initial prototype with regard to affordability, image quality, and adjustability of position and angle. The lack of 3D perception remains the main limitation that remains to be solved. Comparative analyses and larger patient cohorts are needed to assess the performance of the low-cost exoscope compared to conventional visualization platforms.
